# Mental health outcomes in HIV and childhood maltreatment: a systematic review

**DOI:** 10.1186/2046-4053-1-30

**Published:** 2012-06-28

**Authors:** Georgina Spies, Tracie O Afifi, Sarah L Archibald, Christine Fennema-Notestine, Jitender Sareen, Soraya Seedat

**Affiliations:** 1 South African Research Chairs Initiative (SARChI), PTSD program, Francie van Zijl drive, Department of Psychiatry, University of Stellenbosch, Cape Town, 7505, South Africa; 2 MRC Unit on Anxiety and Stress Disorders, Francie van Zijl drive, Department of Psychiatry, University of Stellenbosch, Cape Town, 7505, South Africa; 3 Departments of Psychiatry, Psychology, and Community Health Sciences, Bannatyne Avenue, University of Manitoba, Winnipeg, R3E 0W5, Canada; 4 Department of Community Health Sciences, Bannatyne Avenue, University of Manitoba, Winnipeg, R3E 0W5, Canada; 5 Department of Psychiatry, Gilman drive, University of California San Diego, La Jolla, CA, 92093, USA; 6 Department of Radiology, Gilman Drive, University of California San Diego, La Jolla, CA, 92093, USA

**Keywords:** AIDS, Anxiety, Childhood maltreatment, Depression, HIV, Psychiatric morbidity, Substance abuse

## Abstract

**Background:**

High rates of childhood maltreatment have been documented in HIV-positive men and women. In addition, mental disorders are highly prevalent in both HIV-infected individuals and victims of childhood maltreatment. However, there is a paucity of research investigating the mental health outcomes associated with childhood maltreatment in the context of HIV infection. The present systematic review assessed mental health outcomes in HIV-positive individuals who were victims of childhood maltreatment.

**Methods:**

A systematic search of all retrospective, prospective, or clinical trial studies assessing mental health outcomes associated with HIV and childhood maltreatment. The following online databases were searched on 25–31 August 2010: PubMed, Social Science Citation Index, and the Cochrane Library (the Cochrane Central Register of Controlled Trials and the Cochrane Developmental, Psychosocial and Learning Problems, HIV/AIDS, and Depression, Anxiety and Neurosis registers).

**Results:**

We identified 34 studies suitable for inclusion. A total of 14,935 participants were included in these studies. A variety of mixed mental health outcomes were reported. The most commonly reported psychiatric disorders among HIV-positive individuals with a history of childhood maltreatment included: substance abuse, major depressive disorder, and posttraumatic stress disorder. An association between childhood maltreatment and poor adherence to antiretroviral regimens was also reported in some studies.

**Conclusion:**

A broad range of adult psychopathology has been reported in studies of HIV-infected individuals with a history of childhood maltreatment. However, a direct causal link cannot be well established. Longer term assessment will better delineate the nature, severity, and temporal relationship of childhood maltreatment to mental health outcomes.

## Background

Abuse is a common phenomenon in countries where the prevalence rate of HIV is also high and can include physical, sexual and emotional violence and deprivation or neglect [1]. Studies conducted in developing countries such as South Africa and other African countries have reported high rates of abuse in both adults and children. This includes intimate partner violence (IPV), rape, and childhood abuse or maltreatment [1-3]. Childhood maltreatment has been defined in many different ways. However, for the present review, childhood maltreatment included emotional, physical, and sexual abuse and emotional and physical neglect. According to Bernstein *et al*. [4] sexual abuse is defined as ‘sexual contact or conduct between a child younger than 18 years of age and an adult or older person.’ Physical abuse is defined as ‘bodily assaults on a child by an adult or older person that posed a risk of or resulted in injury.’ Emotional abuse is defined as ‘verbal assaults on a child’s sense of worth or well-being or any humiliating or demeaning behaviour directed toward a child by an adult or older person.’ Physical neglect is defined as ‘the failure of caretakers to provide for a child’s basic physical needs, including food, shelter, clothing, safety, and health care.’ Emotional neglect is defined as ‘the failure of caretakers to meet children’s basic emotional and psychological needs, including love, belonging, nurturance, and support’ [4]. Although women are more vulnerable and regarded as particularly at risk for abuse, men are also victims of rape and childhood maltreatment.

Many studies have investigated the link between adverse childhood experiences such as physical and/or sexual abuse and HIV risk. The experience of childhood maltreatment may increase HIV infection risk indirectly by increasing high-risk behaviors or by interfering with HIV prevention choices [5]. For example, many of the outcomes associated with childhood maltreatment place individuals at increased risk of contracting HIV through behaviors such as transactional sex, unprotected sex, inability to negotiate condom use, alcohol and/or drug abuse, early onset of sexual activities, and multiple sex partners [6-10]. In addition, childhood maltreatment may directly increase the risk of HIV infection through sexual abuse. Injury and the tearing of tissue resulting from sexual violence may increase the likelihood of HIV infection [11]. Studies have also found that childhood maltreatment is strongly associated with adult revictimization which can further increase the risk for HIV among women [5].

The mental health outcomes of HIV-infected individuals have been well documented to date. Research suggests a significant burden of mental illness in individuals living with HIV/AIDS, both globally and in the developing world. Mental illnesses documented in HIV-infected individuals include predominantly substance use, anxiety, and mood disorders [12-19]. Moreover, it has been suggested that HIV disease progression may be hastened by mental disorders such as depression and anxiety [20].

Similarly, research suggests the long-term mental health outcomes of childhood maltreatment include predominantly substance, anxiety, and mood disorders [21,22]. Interestingly, Kaplow and Widom [23] followed 496 individuals with neglect, physical and sexual abuse prior to the age of 12 into adulthood. Their research suggests that an earlier onset of maltreatment predicted more symptoms of anxiety and depression in adulthood, while controlling for gender, race, current age and reports of other abuse. Later onset of maltreatment was predictive of more behavioral problems in adulthood [23]. In a review of child sexual abuse, Johnson [11] outlines a number of child and adult psychological and behavioral consequences of child sexual abuse. These include substance use disorders, and anxiety and mood disorders, amongst others [11].

Although many studies have focused on mental health outcomes in childhood maltreatment and HIV separately, there is a paucity of research investigating childhood maltreatment and HIV in combination, and the associated mental health outcomes in dually affected men and women. HIV-infected women may face more current and past negative life events than men in developing parts of the world [13] and this may lead to significant adult psychopathology and poor adherence to antiretroviral medications [24,25]. In light of this, it is evident that HIV-positive individuals, women in particular, are vulnerable to risk factors associated with abuse, and abuse-related changes in behavioral functioning, which may complicate HIV infection. A systematic assessment and summary of the available evidence is therefore warranted in order to add to the available evidence for both clinical and research decision making.

## Methods

### Search strategy and selection criteria

**Figure 1:**
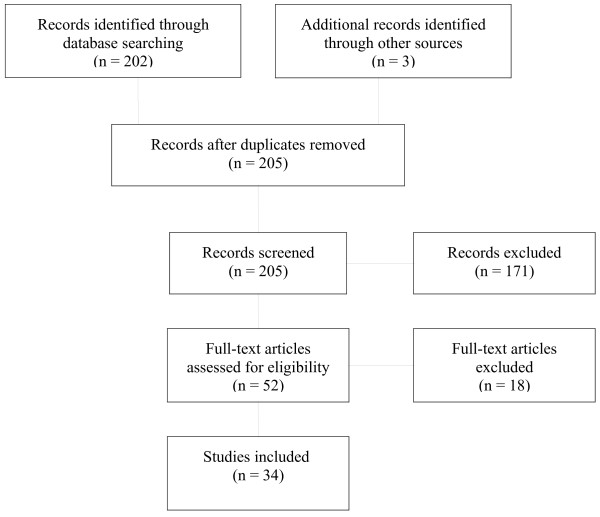
Flow diagram of review process.

We searched the electronic databases PubMed, Social Science Citation Index, the Cochrane Library (The Cochrane Central Register of Controlled Trials: CENTRAL) and the Cochrane Developmental, Psychosocial and Learning Problems, HIV/AIDS, and Depression, Anxiety and Neurosis registers on 25–30 August 2010. No limit on the time period was applied to the search in order to avoid omission of relevant studies. Reference lists of articles identified through database searches and bibliographies of systematic and non-systematic review articles were examined to identify further relevant studies. We included all English language, original research (retrospective and prospective studies) and clinical trials reporting mental health outcomes of childhood trauma in HIV-positive individuals. The population included adult men and women already infected with HIV/AIDS who experienced childhood maltreatment prior to 18 years of age. We excluded systematic and non-systematic review articles and studies of no direct relevance to the comprehensive search. The PubMed search included the following terms: childhood abuse AND HIV. The full search details are as follows: ((‘childhood’[Journal] OR ‘childhood’[All Fields]) AND (‘substance-related disorders’[MeSH Terms] OR (‘substance-related’[All Fields] AND ‘disorders’[All Fields]) OR ‘substance-related disorders’[All Fields] OR ‘abuse’[All Fields])) AND (‘hiv’[MeSH Terms] OR ‘hiv’[All Fields]). No filters were included to ensure that all relevant papers were retrieved. The PubMed search selected those studies that addressed childhood abuse and HIV in all fields. An initial search of titles was undertaken by the reviewer (GS). Studies were included irrespective of sample size and period of follow-up. Titles and abstracts of studies that appeared relevant were then assessed to determine whether they met the inclusion criteria. Abstracts that did not meet the inclusion criteria were rejected. The reviewer assessed full texts of articles that appeared to meet the inclusion criteria of the present study. Information was extracted regarding population characteristics and sample size, study design, outcomes measured and results. No exploration of publication bias was undertaken and it was not possible to conduct a sensitivity analysis for the current review article due to the fact that no meta-analyses were conducted (see Figure 1).

## Results

All databases searched yielded abstracts, and there were duplicates between the databases. All the studies had published results in peer-reviewed journals. Two hundred and five abstracts were identified and reviewed. Of the 205 abstracts identified, 171 articles were excluded. Studies were excluded if they were: of no relevance to the present review, systematic or non-systematic review articles, or not conducted in the population of interest. Titles and abstracts of 52 studies that appeared relevant were then assessed to determine whether they met the inclusion criteria. Of the relevant studies reviewed, 34 articles met inclusion criteria. Three of the 34 articles were sourced from reference lists of other manuscripts. Full text articles for all 34 studies were accessed and reviewed. The reviewed articles are summarized in Table 1. There was heterogeneity in sample characteristics, study methodologies and outcome measures among all studies reviewed in this article. Moreover, some studies included an HIV comparison group whereas others did not, further limiting comparability. For these reasons, it was decided that a meta-analysis of these data was not feasible. Variability in measurement of mental health impairment was noted. Psychiatric symptoms and disorders were assessed according to standard diagnostic criteria, using a structured clinician administered interview and/or through self-report (see Table 1). Although some studies differentiated symptoms and diagnoses, others reported more global levels of psychological distress. For example, two articles sourced reported on global psychological distress and mental health in general, without delineating whether symptoms were depressive in nature or anxiety related, for example [26,27]. Furthermore, some studies simply stated the percentage of HIV-positive maltreatment victims reporting symptoms of anxiety. Although these studies reported global anxiety levels, they failed to differentiate by diagnosis [7,28-30].

**Table 1:** Summary of 34 articles selected for review

**First author [Reference number]**	**N (HIV +/−)**	**Setting and main characteristics of population**	**Type of study**	**Study design**	**Methods (measurement of exposure and outcomes)**	**Summary of outcomes**	**Childhood maltreatment**	**Mental health outcomes**	**High-risk behaviors and revictimization**	**Treatment adherence**
^(1)^ Masten (2007) [31]	49 (49/0).	USA.49 bisexual HIV-positive men with childhood sexual abuse (CSA) histories. The majority were African-American men.	Intervention study.	Baseline survey for a coping group intervention trial.	Participants were individually screened at baseline using a structured clinical interview assessing: demographics, sexual abuse history, depression, posttraumatic stress and risk to self or others.	(1) Full criteria for posttraumatic stress disorder (PTSD).	All participants reported some form of sexual abuse history before the age of 18, with 90% reporting unwanted penetrative anal sex. The average participant age at first abuse was 8.9 years. Most reported more than one abusive experience and frequently had a prolonged abuse exposure.	A total of 21 men (42.9%) met criteria for PTSD.		
^(2)^ Mimiaga (2009) [8]	4295 (258/4037).	USA.4295 men who have sex with men (MSM) enrolled in the EXPLORE study.	Intervention study.	Longitudinal research design. Intervention lasted 48 months with assessments every 6 months.	HIV infection was the primary efficacy outcome. Abuse histories, drug and alcohol use and other psychosocial factors were assessed. A shortened version of the Center for Epidemiologic Studies Depression Scale (CES-D) assessed depression.	(1) Depression. (2) Drug use.	39.7% had a history of CSA.	Participants with a history of CSA were at increased risk for HIV infection over study follow-up. Among participants reporting CSA, the EXPLORE intervention had no effect in reducing HIV infection rates. Participants reporting CSA were significantly more likely to have symptoms of depression and use nonprescription drugs.		
^(3)^ Sikkema (2008) [32]	247 (247/0).	USA. 130 women and 117 men with a history of CSA. All men reported having sex with men.	Intervention study.	Randomized controlled behavioral intervention trial with 12-month follow-up.	A structured interview assessed depression, PTSD, and risk to self or others. A modified and expanded version of the Traumatic Experiences Questionnaire (TEQ) assessed exposure to traumatic events, including sexual abuse during childhood, and adulthood.	(1) Sexual revictimization. (2) PTSD.	The average age of first abuse was 8.8 years. Most (90%) experienced penetrative vaginal or anal sexual abuse as a child or adolescent; 87% experienced sexual revictimization, with more than half of those revictimized as children or adolescents. Only 10% of participants reported a single episode of abuse. On average, CSA lasted 4 years and participants had 2 abusers (only 39% of participants reported 1 abuser).	40% of the sample met diagnostic criteria for PTSD.		
^(4)^ Sikkema (2004) [33]	28 (28/0).	USA. Twenty-eight HIV-positive participants (7 men and 21 women).	Intervention study.	Baseline survey for a coping intervention trial.	Trauma Symptom Checklist: childhood and adult traumatic experiences. Personality Assessment Inventory: self-administered objective inventory of adult personality. Trauma Symptom Inventory (TSI): acute and chronic posttraumatic symptomatology.	(1) Mood disorders.(2) Anxiety disorders, including PTSD.(3) Substance abuse.(4) Personality disorder.(5) Revictimization	Prior to age 12, 71.4% reported oral sexual abuse and 85.7% reported penetrative anal sexual abuse. During adolescence, 57.1% of the men experienced some form of sexual abuse. 76.2% of women reported unwanted touching or fondling, 25% reported oral sexual abuse, and 57.1% reported penetrative anal or vaginal sexual abuse during childhood (age 12 or younger). During adolescence, 85.7% unwanted touching or fondling, 57.1% oral sexual abuse, and 81.0% penetrative anal or vaginal sexual abuse.	85% of the participants received an indicator of an Axis I diagnosis: the most frequent diagnostic categories were mood disorders (46.4%), anxiety disorders, including PTSD (32%), and substance abuse (25%). On Axis II, 28.5% received at least one diagnostic indicator of a personality disorder.		
^(5)^ Sikkema (2007) [34]	198 (198/0).	USA. 107 women and 91 men with CSA. All men reported having sex with men.	Intervention study.	Baseline survey for a coping intervention trial.	Depressive symptomatology: Beck Depression Inventory (BDI). The Impact of Events Scale: PTSD symptoms.	(1) PTSD.	89% of participants experienced penetrative anal or vaginal abuse during childhood or adolescence. Fifty-five percent of participants reported sexual abuse during both childhood and adolescence.	40% of study participants met DSM-IV diagnostic criteria for PTSD.		
^(6)^ Williams (2008) [35]	137 (137/0).	USA. 137 HIV-positive gay and non-gay identifying African-American and Latino men with histories of CSA.	Intervention study.	Randomized clinical trial compared the effects of two 6-session interventions.	A randomized clinical trial aimed at decreasing high-risk sexual behaviors, number of sexual partners, and depressive symptoms. The CES-D assessed depression.	(1) High levels of depression at baseline.(2) Significant decrease in depression from 3 to 6 months follow-up.	Histories of CSA.	There were high levels of depression at baseline, M = 23. There was a significant decrease in depressive symptoms from the 3 month to the 6 month follow-up assessment for the sample as a whole (M = 22.42, for 3 months depression and M = 20.66 for 6 months depression).		
^(7)^ Holmes (1997) [36]	95 (95/0).	USA. 95 HIV seropositive men. The majority were Caucasian men (67%). Sexual practices were homosexual/bisexual in 87 (92%) participants.	Survey HIV + only	Quantitative cross-sectional survey design.	Sociodemographic and sexual abuse histories were obtained. The Structured Clinical Interview for the DSM Disorders) was used to identify Psychoactive Substance Use Disorder (PSUD).	(1) PSUD. (2) Increased risk of intravenous drug use (IVDU).	Nineteen (20%) participants had sexual abuse histories. First abuse occurred at a mean age of 8.1 years.	Fifty-five (58%) participants met the criteria for a diagnosis of PSUD at some time in their lifetime and nine (9%) currently met diagnostic criteria. Men with histories of sexual abuse did not exhibit a significantly increased risk of lifetime or current PSUD. When rates were examined by type of administration method, men with reported histories of sexual abuse did show a significantly increased risk of lifetime IVDU.		
^(8)^ Myers (2006) [37]	147 (147/0).	USA. 147 HIV-positive women. The majority were African-American (*n =* 79).	Survey HIV + only	Baseline survey for a risk reduction intervention trial.	CSA: revised Wyatt Sex History Questionnaire (WSHQ-R). PTSD: PTSD diagnostic module of the University of Michigan version of the Composite International Diagnostic Interview. Trauma-related sexual symptoms were assessed with the TSI. Depression was assessed with the CES-D.	(1) More PTSD symptoms in those abused by a family member or by both a family and non-family member.	18% of the women reported one or more less severe sexual abuse incidents. 40% experienced one severe incident, including attempted or completed oral, anal, or vaginal sex, and digital penetration. 18% experienced one severe and one or more less severe incidents, and 24% experienced two or more severe incidents. 34% of the women reported being abused by a perpetrator that was not a family member. 43% reported abuse by a family member, and 24% experienced being abused by both intra- and interfamilial perpetrators. The mean number of sexual abuse incidents was 1.8, with a range of 1 to 6 incidents, and on average, the abuse continued for 2 years.	The relationship to perpetrator was a significant predictor of PTSD symptoms, with more PTSD symptoms among those who reported intrafamilial abuse or both intrafamilial and extrafamilial abuse compared with those who reported only experiencing extra familial abuse.		
^(9)^ Roy (2003) [38]	149 (149/0).	USA. 149 HIV positive substance-dependent patients. There were more males than females in the sample and the majority were African-American.	Survey HIV + only	Quantitative cross-sectional survey design.	Structured Clinical Interview for DSM-IV: depression. The Eysenck Personality Questionnaire (EPQ) assessed neuroticism, extraversion, and psychoticism. The Childhood Trauma Questionnaire (CTQ) assessed for childhood abuse and neglect.	(1) Suicidality(2) Substance dependence.(3) Lifetime depressive disorder.4) High neuroticism scores.	HIV positive patients who had attempted suicide had significantly higher scores on the CTQ for childhood emotional abuse, physical abuse, sexual abuse, emotional neglect and physical neglect.	66 (44.3%) had attempted suicide and 83 (55.7%) had not. Significantly more of those who had attempted suicide were female. Of the 66 patients, 51 who had attempted suicide had a primary substance dependence diagnosis. Significantly more of the patients who had attempted suicide had a lifetime history of a depressive episode. HIV positive attempters also had significantly higher neuroticism scores on the EPQ.		
^(10)^ Allers (1999) [39]	52 (52/0).	USA. 52 HIV-positive individuals. Of the 45 male and 7 females, 36 were White and 16 were Black.	Survey HIV + only	Qualitative survey design.	A semi-structured interview conducted by male HIV counselors. This interview tapped into variables such as: history of childhood abuse, pre-HIV histories of abusive or revictimizing relationships or both, depression, sexual compulsivity and alcohol or other drug abuse.	(1) History of alcohol or drug abuse.(2) Chronic depressive symptomatology.(3) Revictimization.(4) Sexually compulsive behaviors.	A total of 65% (*n =* 34) reported a history of childhood sexual or physical abuse or both. 35.3% (*n =* 12) reported physical abuse only, and 64.7% (*n =* 22) reported sexual abuse. All 22 participants reporting sexual abuse also reported some additional form of childhood physical abuse. Of these participants, 88% (*n =* 30) reported a history of alcohol or other drug abuse, 82% (*n =* 28) reported revictimizing relationships, 68% (*n =* 23) reported chronic depressive symptomatology, and 50% (*n =* 17) reported engaging in sexually compulsive behaviors.	Of these participants, 88% (*n =* 30) reported a history of alcohol or other drug abuse, and 68% (*n =* 23) reported chronic depressive symptomatology.	82% (*n =* 28) reported revictimizing relationships, and 50% (*n =* 17) reported engaging in sexually compulsive behaviors.	
^(11)^ Brennan (2007) [40]	936 (936/0).	USA. 936 gay and bisexual men. The majority (95.3%) were gay and White (88.8%) men.	Survey HIV + only	Quantitative cross-sectional survey design: retrospective data.	A self-administered survey investigating: HIV/sexually transmitted infection (STI) status, self-defined current use of sex-related drugs, other HIV risk behaviors and history of CSA.	(1) Current drug abuse.(2) Transactional sex practices.	15.5% (*n =* 134) of survey respondents reported a history of CSA. Those who reported experiencing abuse regularly were more likely to be HIV positive.	Those who reported experiencing abuse regularly were more likely to be a current user of sex-related drugs.	Those who reported experiencing abuse regularly were more likely to have exchanged sex for payment, and be a current user of sex-related drugs.	
^(12)^ Clum (2009) [41]	40 (40/0).	USA. 40 young HIV-positive women recruited from HIV clinics.	Survey HIV + only	Mixed method design (qualitative and quantitative surveys).	A modified version of the Life Story Interview was used to cover abuse experiences, cognitive and emotional consequences of abuse, coping strategies, and sexual behavior and relationships. PTSD symptoms were assessed with an interviewer-administered Posttraumatic Diagnostic Scale.	(1) PTSD symptomatology ranging from mild to severe.(2) Reported difficulties in sexual, family, and friend relationships, general life satisfaction, and leisure time activities.(3) Substance abuse.	75% of the women reported sexual abuse, 80% reported physical abuse, and 55% reported both types of abuse.	The average PTSD score was 20.75, reflecting moderate to severe levels of PTSD symptoms. 15% of the sample reported mild PTSD symptoms (<10), 37.5% reported moderate symptoms (10 to 20), 30% reported moderate to severe symptoms (21 to 35), and 15% reported severe symptoms (>35). Avoidance and substance use were frequently utilized as coping strategies.		
^(13)^ Cohen (2004) [25]	1165 (1165/0).	USA. 1165 HIV-positive women. 635 participants were using highly active antiretroviral therapy (HAART), 254 participants not using HAART although it had been indicated and 276 participants not on HAART which had not been indicated.	Survey HIV + only	Quantitative survey design.	A standardized interview-based survey assessed demographics, medical and psychosocial history, history of cigarette smoking, alcohol use, illicit drug use and drug treatment programs, sexual history and history of medication use, and reasons for non-adherence at each 6-month visit. The CES-D measured depressive symptoms. Women were also asked questions about physical, sexual, or emotional coercion.	(1) Poor treatment adherence.(2). Drug abuse.(3) High levels of depression in all groups.	72% of women using HAART reported a history of physical or sexual abuse. For women who were not using HAART, 80% reported a history of physical or sexual abuse. Current crack, cocaine, or heroin use, being non-White, and experiencing any physical or sexual abuse increased the likelihood of no HAART use.	A lower percentage of women with a history of past and current use of crack, cocaine, or heroin were using HAART. Women in the groups did not differ significantly in having high levels of depressive symptoms.		A lower percentage of women with a history of physical or sexual abuse reported using HAART. Women who used crack, cocaine, or heroin in the past year were more than twice as likely to report lack of HAART use, even when indicated. Similarly, women with a history of any physical/sexual abuse were more than 1.5 times more likely to lack HAART when clinically eligible.
^(14)^ Gielen (2001) [26]	287 (287/0).	USA. 287 HIV-positive women. 94% of the women were African-American.	Survey HIV + only	Quantitative cross-sectional survey design.	Health status and quality of life were evaluated with a modified version of the Medical Outcomes Study HIV Health Survey. HIV-related characteristics, social support and health promoting behaviors were assessed. Adult violence history and whether they had ever been sexually abused or raped as a child was assessed using a dichotomous response variable (yes/no).	(1) Drug abuse.(2) Poor mental health, physical functioning, and quality of life.(3) Social networks and health promoting behaviors improved mental health.	A history of CSA was reported by 41% of the sample.	55% had a history of injection drug use. Women with a history of child sexual abuse reported significantly lower scores on measures of mental health, physical functioning, and quality of life. Women with larger social networks and who practiced more health promoting behaviors reported higher levels of mental health, whereas those who had been sexually abused as children reported significantly worse mental health.	63% reported having been physically or sexually assaulted at least once as an adult.	
^(15)^ Henny (2007) [42]	644 (644/0).	USA. HIV-seropositive homeless or unstably housed adults (*n =* 644). The sample included 15 male-to-female transgender people	Survey HIV + only	Quantitative cross-sectional survey design.	Dichotomous variables (yes/no) assessed adult and childhood abuse, and current and lifetime illicit drug use. The CAGE questionnaire investigated alcohol use. Depressive symptoms were measured by the CES-D. Self-perception of stress was measured using the Perceived Stress Scale.	(1) Alcohol abuse.(2) Depressive symptomatology.(3) Transactional sex.	80.3% of the sample reported a history of any physical or sexual abuse. 53% reported childhood physical abuse and 38.7% reported CSA. Victims of CSA were nearly three times as likely to be female.	Victims of childhood physical abuse were more likely to have abused alcohol. Persons experiencing childhood physical abuse also were twice as likely to report symptoms indicating depression.	Persons experiencing childhood physical abuse also were twice as likely to have ever exchanged sex for money, drugs, or shelter.	
^(16)^ Kalichman (2002) [28]	357 (357/0).	USA. 357 men and women living with HIV/AIDS. Study participants were 242 (68%) men, 110 (31%) women, and 5 (1%) transgender persons. The majority of the sample was African-American (76%).	Survey HIV + only	Quantitative cross-sectional survey design.	A dichotomous variable (yes/no) assessed sexual abuse history and substance abuse history. Trauma indicators were adapted from diagnostic symptoms of PTSD. Symptoms of depression were assessed with the CES-D. The Trait-Anxiety Scale assessed anxiety. A 6-item scale to assess pessimism was developed. Symptoms of obsessiveness–compulsiveness were assessed using six items from the Obsessive–Compulsive Scale of the schedule for nonadaptive personality (SNAP). Similarly, six items from the Borderline Personality Scale of the SNAP were used to assess borderline personality characteristics.	(1) Substance abuse.(2) Anxiety symptoms.(3) Depression symptoms.(4) Borderline personality symptoms.(5) Current PTSD symptoms.(6) Trauma symptoms correlated with the number of sexual assaults reported.	68% of women and 35% of men living with HIV/AIDS reported a history of sexual assault since age 15.	History of sexual assault was related to history of substance use and mental health treatment. Sexual assault survivors reported greater anxiety, depression, and symptoms of borderline personality than persons who had not been sexually assaulted. Persons who reported having been sexually assaulted reported current trauma symptoms. Specifically, 24% stated that they think of the experience on a regular basis, 20% have nightmares about the experience, 60% reported that the experience affects them today, and 47% stated that the experience interferes with their relationships. Number of trauma symptoms correlated with the number of sexual assault experiences reported.		
^(17)^ Kang (2008) [43]	220 (220/0).	USA. All participants were HIV-positive heroin and/or crack cocaine using African-Americans or Hispanics. There were 146 males and 74 females.	Survey HIV + only	Baseline survey for an intervention study.	Childhood abuse experience: CTQ. Depression: CES-D. Health status items included: general health rating and HIV-related symptoms. Lifetime medical conditions were also examined.	(1) Alcohol and drug abuse.(2) High depression levels.(3) Poor treatment adherence.	Women were more likely to report CSA (51% versus 39%) and childhood physical abuse (64% versus 54%).	Men were more likely to use alcohol to intoxication and currently inject drugs, and females were more likely to use crack. Both men and women had high depression levels. 81% of women and 76% of men had a score of 16 or higher on the CES-D.		For both men and women, use of HIV medications was negatively associated with CSA experience.
^(18)^ Kimerling (1999^a^) [44]	67 (67/0).	USA. Sample included 67 African-American HIV-infected women beyond the initial stages of HIV infection.	Survey HIV + only	Longitudinal design: 12–14 months apart, with an average time of 13.4 months apart.	Life Stressor Checklist: identify life stressors with greater prevalence for women. Impact of Events Scale-Revised: the presence and intensity of PTSD symptoms.	(1) PTSD (both symptom clusters and full criteria).	62% of the sample reported experiencing at least one traumatic event. 30% of the sample experienced completed rape and 33% experienced physical assault. These both included rape and assault as a child.	The majority who met the stressor criterion also met criteria for at least one other symptom cluster for PTSD, whereas 35% of the sample met full criteria. 88% of the sample met criteria for the re-experiencing symptom cluster, 74% for the avoidance/numbing symptom cluster and 70% for the hyper arousal symptom cluster.		
^(19)^ Martinez (2002) [45]	41 (41/0).	USA. 41 HIV-positive women. The majority of the sample (51%) was African-American.	Survey HIV + only	Quantitative cross-sectional survey design: retrospective data.	The Life Stressor Checklist-Revised was completed in order to examine the frequency and types of traumatic life events. The PTSD Checklist-Civilian Version 29 was used to assess current PTSD symptoms.	(1) Partial and full PTSD.(2) Level of PTSD significantly related to number of life events experienced and perceived social support.	61% of women had experienced growing up with violence in the home. 59% were emotionally abused or neglected. 32% had been abused or physically attacked by a known person before the age of 16. Similarly, 32% were sexually touched or made to touch someone before age 16 and 31% were forced to have some type of sex before age 16.	42% of the HIV-positive women were likely to meet criteria for full current PTSD and an additional 22% for partial PTSD. Women reported having experienced a mean of 12 traumatic life events. The level of PTSD was significantly related to the number of life events experienced and to perceived social support from friends and family.		
^(20)^ Martinez (2009) [46]	174 (174/0).	USA. HIV-positive youth enrolled in a young adult HIV clinic between 1998 and 2006. 58 were females and 116 were males. The majority (79%) were African-American.	Survey HIV + only	Quantitative cross-sectional survey design.	Client Diagnostic Questionnaire was used to screen for mental health disorders and violence. All youth subsequently had diagnostic interviews conducted by psychologists.	(1) Major depressive disorder (MDD).(2) Generalized anxiety disorder.(3) PTSD.(4) Alcohol and substance abuse disorders.	Violence reported included physical abuse (24% in childhood; 19% in adolescents), sexual abuse (28% in childhood; 15% in adolescents), dating violence (18%), and family violence (44%). Females had higher sexual abuse (P < .001).	Psychological disorders included: MDD (15%), generalized anxiety disorder (17%); PTSD (28%); alcohol abuse disorder (19%); and substance abuse disorder (31%). Physically abused youth had higher symptoms of anxiety and PTSD. Sexually abused youth had higher symptoms of PTSD (*P* < 0.05). Youth with family violence had higher symptoms of Anxiety Disorder (*P* < 0.05) and PTSD (*P* < 0.01).		
^(21)^ McKeown (2003) [47]	20 (20/0).	Canada. 20 HIV-positive women. Eighteen (90%) self-identified as aboriginal.	Survey HIV + only	Qualitative research design.	Open ended interviews were conducted to obtain information on childhood and adulthood experiences.	(1) Drug abuse as coping strategy.(2) Transactional sex.(3) Past suicide attempts.(4) Reported diagnoses of MDD, PTSD, schizophrenia, panic disorder, and multiple personality disorder.	Women who had experienced CSA.	A few of the women recounted past attempts of suicide. A number of women, at the time of the interview, reported a diagnosis of mental illness including depression, multiple personality disorder, panic attacks, PTSD and schizophrenia. Most participants reported IVDU on a regular basis in the past, with one reporting current use of IV drugs.	The majority who experienced CSA reported involvement in the sex trade and drug abuse as economic and emotional survival/coping strategies.	
^(22)^ Meade (2009) [7]	271 (271/0).	USA. 271 HIV-positive individuals with histories of CSA. 50% female and 69% African-American. The men were primarily (94%) gay/bisexual.	Survey HIV + only	Baseline survey for a coping intervention trial	A modified version of the TEQ was used to verify childhood abuse history. The BDI was used to identify severe depression.	(1) Depressive disorder.(2) Anxiety disorder.(3) Psychotic disorder.(4) Adjustment disorder.(5) Bipolar disorder.(6) Alcohol and drug abuse.(7) Undergone mental health treatment.	271 HIV-positive individuals with histories of CSA.	Approximately half of the sample (53%) screened positive for one or more psychiatric disorders (30% depressive, 25% anxiety, 11% psychotic, 10% adjustment, 4% bipolar). Approximately one third (37%) used illicit drugs and 10% reported binge drinking in the past 4 months. Many participants also received mental health treatment in the past 4 months. Those screening positive for a psychiatric disorder were more likely than those who did not to have received mental health treatment (59% versus 41%).		
^(23)^ Pence (2007) [29]	611 (611/0).	USA. 611 HIV-infected individuals. Sixty four percent of participants were African-American and 31% were female.	Survey HIV + only	Quantitative cross-sectional survey design.	Patients completed the Brief Symptoms Inventory (BSI), an assessment of current psychological symptoms. Substance use was measured with the Addiction Severity Index. PTSD symptoms were assessed with the PTSD Checklist.	(1) PTSD.(2) More than half had a probable psychiatric disorder on the BSI.(3) High levels of depression.(4) High levels of anxiety.(5) Substance abuse.	Most respondents (91%) reported experiencing at least one traumatic event in their lifetime. 30.4% experienced CSA and 20.6% severe physical abuse as a child.	16% of the sample met criteria for PTSD, 53.9% of the sample had a probable psychiatric disorder on the BSI. 34.7% of the sample had depressive symptoms above the 90th percentile and 29.5% had anxiety symptoms above the 90th percentile. 22.3% of the sample was engaging in any non-marijuana substance abuse and 20% were using multiple substances.		
^(24)^ Sikkema (2009) [48]	256 (256/0).	USA. 256 HIV-positive adults with CSA histories. There were 132 women and 124 MSM. The majority (67.3%) was African-American.	Survey HIV + only	Quantitative cross-sectional survey design.	A modified version of the TEQ assessed abuse history. Depression and suicidal ideation: BDI. TSI: PTSD. Substance abuse and sexual behavior were also assessed using self-developed screening tools.	(1) Sexual revictimization.(2) Mood and anxiety symptoms.(3) PTSD symptoms.(4) Alcohol and drug use.	All participants reported abuse histories. 90% had experienced penetrative vaginal or anal sexual abuse as a child or adolescent.	The mean score for mood and anxiety symptoms was 29.8 in women and 28.2 in men. Mean score for trauma-related symptoms was 40.4 in women and 28.9 in men. Alcohol use in the past 4 months was 31.8% in women and 53.2% in men. Marijuana use in the past 4 months was 18.2% in women and 36.3% in men. Cocaine and/or Crack use in the past 4 months was 18.9% in women and 33.1% in men.	87% experienced sexual revictimization at some point in their lives.	
^(25)^ Simoni (2000) [49]	230 (230/0).	USA. Sample consisted of 230 HIV-positive women. The majority (46%) described themselves as African-American.	Survey HIV + only	Quantitative cross-sectional survey design.	Demographics, trauma, coping strategies and current depressive symptomatology were assessed. Respondents completed the CES-D. Self-reported trauma histories were documented.	(1) High scores of depressive symptoms.(2) Positive correlation between childhood abuse and current adaptive and avoidant coping strategies.(3) Avoidant coping was strongly associated with CES-D scores.	A high prevalence of abuse in childhood (50%) and adulthood (68%); 7% reported physical assault or rape in the last 90 days.	Childhood abuse was significantly correlated with both adult and recent trauma, and each type of trauma correlated with CES-D scores. The mean CES-D score was 22.49; 66% had a sum score of 16 or above, indicative of possible clinical depression.		
^(26)^ Tarakeshwar (2005) [50]	28 (28/0).	USA. 28 HIV-positive women with CSA histories. The majority were African-American (67.9%).	Survey HIV + only	Qualitative research design.	A clinical psychologist and a social worker conducted in-depth qualitative interviews. The interview was developed on the basis of the published literature and the goal of developing a coping-focused intervention for women with CSA history and HIV. The interview protocol used a semi-structured interview format that addressed the impact of sexual abuse and HIV on their life and the ways they coped with these traumas.	(1) Reported cumulative trauma-related distress.(2) Current use of psychiatric medications for: depression, anxiety (agoraphobia, panic disorder), PTSD.(3) Frequent hospital visits for physical complaints.(4) Substance abuse.(5) Revictimization.	78.6% of the sample revealed unwanted touching or fondling, 57.1% reported sexual intercourse, and 57.1% were asked to engage in sexual acts under verbal and emotional pressure (before 13). During adolescence (13–17 years), their reports of unwanted sexual abuse experiences increased: 82.1% for intercourse, 64.3% for oral sex, 71.4% for forced or threatened sexual acts, 75.0% for verbal and emotional pressure, and 35.7% for unwanted sexual acts that occurred when they had passed out or were drunk or asleep. Many (40%) of the women were abused by family members.	Most of the women reported having encountered multiple traumatic experiences and reported cumulative distress as a result of these experiences. Many were using psychiatric medications for symptoms of depression, anxiety (e.g., agoraphobia, panic disorder), and PTSD. PTSD such as flashbacks and hyper vigilance around places and occasions that reminded them of their sexual abuse were common. A few women stated that their distress led to frequent visits to the hospital for physical complaints as they psychologically struggled to comprehend their sexually abusive experiences since childhood. Using illicit substances (e.g. drugs) helped all the women numb their symptoms of emotional distress and feelings of anger and betrayal generated by their CSA.	75% reported sexual revictimization.	
^(27)^ Tarakeshwar (2006) [51]	266 (266/0).	USA. 266 HIV-positive participants. There were 133 males, 129 females, and 4 transgender. The majority (71.5%) was African-American.	Survey HIV + only	Quantitative cross-sectional survey design.	Participants were screened for abuse histories in childhood, adolescence, and adulthood. The BDI was used to assess depressive symptomatology. Perspectives on addressing trauma symptoms, HIV-related stress, and resiliency were also assessed using self-developed screening tools and modified scales.	(1) Substance use treatment in the past four months.(2) Lower resiliency and greater HIV-related stress was related to negative feelings about addressing trauma.(3) Revictimization.	91% of the participants had been sexually abused as children, 77% had been abused during adolescence. 71.5% of men and 66.7% of women reported unwanted vaginal or anal sex in childhood.	54% of men and 52% of women had at least one visit to a mental health provider in past 4 months. 39.5% of men and 38% of women were on psychiatric medications. Substance use treatment in the past 4 months was reported in 38.8% of men and 29.5% of women.	56% had been sexually revictimized as adults.	
^(28)^ Welles (2009) [30]	593 (593/0).	USA. 593 HIV + MSM.	Survey HIV + only	Baseline survey for a risk reduction intervention trial.	Participants reported the frequency of CSA. Brief Symptom Checklist was used to assess depression and anxiety.	(1) High levels of depression.(2) High levels of anxiety.(3) Reported current and lifetime alcohol and drug problems.	Of participants, 47% reported CSA, with 32% reporting CSA occurring often or sometimes. Although most (154 or 58%) reported the gender of the perpetrator as male, 38 (14%) reported CSA by a female, and 75 (28%) by both.	HIV + reporting history of CSA had significantly higher levels of depression and anxiety, with 39% reporting the highest quartile scores for the depression and anxiety inventory, compared with 24% of men reporting no CSA. Men reporting CSA were more likely to believe that they have or had problems with drugs or alcohol.		
^(29)^ Wyatt (2005) [52]	75 (75/0).	USA. 75 HIV-positive women with histories of CSA.	Survey HIV + only	Baseline survey for an intervention trial.	Women were administered the WSHQ-R. Five measures were used to assess patterns of substance abuse.	(1) Substance abuse.(2) Lifetime alcohol or drug treatment.	All women in the sample had a history of CSA.	83% of the sample reported having used at least 1 of 13 substances regularly at some point in their lives. 28% of the sample reported engaging in regular injection drug use. 54% of the women reported having taken part in an alcohol or drug treatment program at some point in their lifetime.		
^(30)^ Paxton (2004) [53]	457 (299/158).	USA. 65.4% of the sample was HIV-seropositive. The majority of the sample was African-American.	General survey (mixed).	Quantitative cross-sectional survey design.	Alcohol and drug abuse/dependence, depression, and panic disorder: subscales of the University of Michigan Revised Short Form of the Composite International Diagnostic Inventory. Posttraumatic stress symptoms: revised 17-item short form clinical checklist. Select items from the WSHQ-R measured exposure to sexual and other lifetime trauma.	(1) PTSD symptoms.(2) Substance abuse.(3) Risky health behaviors.(4) Chronic stress.(5) History of psychiatric disorders.	HIV-positive women were more likely to report a history of CSA.	HIV-positive women with a history of CSA were more likely to report posttraumatic stress, substance abuse, chronic stress, and psychiatric history than HIV-negative counterparts.	HIV-positive women with a history of CSA were more likely to report risky health behaviors than HIV-negative counterparts.	
^(31)^ Cohen (2000) [6]	1645 (1288/357).	USA. 1288 HIV-positive women and 357 HIV-negative women. The majority (64%) were African-American.	General survey (mixed).	Quantitative cross-sectional survey design: retrospective data.	A survey investigating three areas of violence: any domestic violence, recent domestic violence and CSA. Lifetime substance abuse and injection drug use in the past 6 months was assessed. Finally, HIV risk behaviors were assessed.	(1) Drug use.(2) HIV-risk behaviors.(3) Revictimization.	31% of HIV-positive women reported a history of CSA	Women reporting past domestic violence or CSA were more likely than women without such histories to have used drugs at some point in their lives.	Women reporting past domestic violence or CSA were more likely than women without such histories to have had more than 10 lifetime male partners; to have traded sex for money, drugs, or shelter; and to have been forced to have sex with a person known to be HIV positive. Women who reported CSA were more likely to report a lifetime history of domestic violence and to have experienced domestic violence in the past year.	
^(32)^ Kalichman (2004^a^) [3]	272 (6/19),	South Africa. 272 women living with sexual assault histories. Nearly all (99%) of the women were African.	General survey (mixed).	Quantitative cross-sectional survey design.	Self-administered anonymous surveys assessing sexual assault history, substance use, history of HIV risk factors, and sexual behavior.	(1) Alcohol and drug use.(2) Transactional sex	6 women (11%) were HIV-positive and 19 (33%) were HIV-negative. The majority of women (56%) did not know their HIV status. 40% (*N =*/119) of women reported a history of sexual assault. 26 (21%) of the women had experienced sexual assault before the age of 20.		Women who had been sexually assaulted were significantly more likely to have shared injection drug equipment, exchanged sex to meet survival needs, and used alcohol compared to women who had not been sexually assaulted.	
^(33)^ Kalichman (2004^b^) [54]	647 (498/142).	USA. 647 men with CSA histories. The majority were Caucasian (70%).	General survey (mixed).	Quantitative cross-sectional survey design.	Self-administered surveys were used to assess demographics, sexual abuse history, substance use and sexual risk behaviors.	(1) Symptoms of borderline personality disorder.(2) Alcohol and drug abuse.(3) Having undergone treatment for substance abuse.	93 (15%) of the men reported being forced to have sex when they were 16 years or younger by a man at least 5 years older. Of these 93 men, the average age of first abuse was 9.3 years. Sexually abused men were more likely to report childhood physical abuse relative to non-abused men (41% vs. 12%). Men who were sexually abuse were more likely to have tested HIV-positive (40%) relative to non-abused men (19%). 77% of the men were HIV-negative and 22% were (9%).	HIV-positive. Abused men endorsed more symptoms of borderline personality disorder. Contrary to expectations, abused men did not differ in dissociation symptoms or trauma-related anxiety when compared to non-abused counterparts. Abused men were more likely to report alcohol and drug abuse in the past 6 months and having undergone treatment (28%) compared to non-abused men.		
^(34)^ Kimerling (1999^b^) [27]	236 (88/148).	USA. 88 African-American HIV-infected women and 148 uninfected women.	General survey (mixed).	Quantitative cross-sectional survey design: retrospective data.	The Life Stressor Checklist: history of victimization. The BSI: level of general or global distress. The Hamilton Clinician’s Rating Scale for Depression: depression and to serve as a more objective measure of psychological distress.	(1) High levels of global psychological distress.(2) Depression.(3) Greater physical distress and AIDS-defining conditions.	A history of completed rape contributed the greatest risk for HIV infection. Women who reported completed rape identified the worst experience to have occurred at 18.27 years old. This variable included rape as a child.	HIV-infected victims reported higher levels of global psychological distress, and greater severity of clinician-rated symptoms of depression. HIV-infected victims also reported significantly greater distress with physical symptoms and higher rates of AIDS-defining conditions than did non-victims.		

*BDI*, Beck Depression Inventory; *BSI*, Brief Symptom Inventory; *CES-D*, Center for Epidemiologic Studies Depression Scale; *CSA*, childhood sexual abuse; *CTQ*, Childhood Trauma Questionnaire; *EPQ*, Eysenck Personality Questionnaire; *HAART*, highly active antiretroviral therapy; *IVDU*, intravenous drug use; *MDD*, Major depressive disorder; *MSM*, men who have sex with men; *PTSD*, posttraumatic stress disorder; *PSUD*, Psychoactive Substance Use Disorder; *SNAP*, schedule for nonadaptive personality; *STI*, sexually transmitted infection; *TEQ*, Traumatic Experiences Questionnaire; *TSI*, Trauma Symptom Inventory; *WSHQ-R*, revised Wyatt Sex History Questionnaire.

A history of childhood maltreatment was also assessed in different ways, but all studies relied on self-reported history of childhood maltreatment, and most assessments were retrospective in nature. In some studies, childhood maltreatment included various forms/types such as physical abuse and neglect, emotional abuse and neglect, and sexual abuse [43]. Other studies only examined childhood sexual abuse (CSA) [6,26,40] or combined sexual and physical abuse into one category of child abuse [25,39,41]. Some studies utilized validated self-report measures sensitive in tapping into various forms of childhood abuse and neglect [38,43]. A widely used example of such a measure is the Childhood Trauma Questionnaire [4]. However, many studies established a history of childhood abuse by simply asking a single question such as ‘have you ever experienced a sexual assault or rape as a child or teenager, that is, when you were 18 years of age or younger?’ and using a dichotomous response option (Yes/No) [26,28,42].

### Childhood maltreatment

Childhood maltreatment, such as physical and sexual abuse is a common phenomenon in the general population (uninfected individuals). CSA is reported by as many as 32% of women and 14% of men in the general population, whereas physical abuse is experienced by 22% of males and 19.5% of females in the general population [55]. However, rates of childhood maltreatment in HIV-positive individuals are significantly higher, suggesting that the experience of childhood maltreatment in the context of HIV is worthy of greater attention. Rates of CSA among HIV-positive individuals range from 32% to 76%, respectively [28,56,57].

### Mental health outcomes

In reviewing the articles, a wide range of mental health symptoms and disorders were reported. The most commonly reported psychiatric symptomatology among HIV-positive individuals with a history of childhood maltreatment included (study number in Table 1): drug and/or alcohol abuse/dependence (2,4,7,9-17,20-24,26-33), depression (2,4,6,9,10,13,15,17,20-26,28) and posttraumatic stress disorder (PTSD) (1,3-5,8,12,18-20,23,24,26,30). Other mental health outcomes reported included (reference number in Table 1): anxiety (4,16,22-24,26,28), generalized anxiety disorder (20), borderline personality (16,33), panic disorder (21,26), agoraphobia (26), schizophrenia (21), psychotic disorder (22), adjustment disorder (22), bipolar disorder (22), suicidality (9,21), neuroticism (9), personality disorder (4) and multiple personality disorder (21). Moreover, when examining mental health outcomes such as drug abuse and depressive symptomatology, two articles also reported an association between childhood maltreatment and poor treatment adherence to antiretroviral regimens (13,17). Physical complaints/distress and reduced quality of life was also a finding in the studies reviewed (14,18,26). Findings from several studies indicated that participants had at some time in their lives undergone mental health treatment (22,26,27,29,33). Many studies found participants commonly reporting engagement in high-risk behaviors such as transactional sex or compulsive sexual behaviors (10,11,15,21,30-32) and adult revictimization was common (3,4,10,24,26,27,31). Individual rates of psychopathology reported in studies varied. The percentage of participants in individual studies who received a diagnosis of PTSD included (study number in Table 1): 42.9% (1), 40% (3), 32% (4), 40% (5), 35% (18), 42% (19), 28% (20) and 16% (23). Other studies reported PTSD scores on self-report/interviewer administered instruments. The average PTSD score on the Posttraumatic Diagnostic Scale was 20.75 in one study, with 30% of participants reporting moderate to severe symptoms and 15% reporting severe symptoms (12). In another study, the mean score on the Trauma Symptom Inventory for trauma-related symptoms was 40.4 in women and 28.9 in men (24). PTSD was not an inclusion criterion in the research study but rather an unselected observation for most studies (1,3,5,12,20,23,24). However, in one study, participants were only included if there was evidence of psychological distress or if criteria for mood or anxiety disorders were met (4).

The percentage of participants in individual studies who received a diagnosis of mood disorders included (study number in Table 1): 46.4% (4) 68% (10), 15% (20), 30% (22), 34.7% (23) and 39% (28). Other studies reported depression scores on self-report/interviewer administered instruments. The mean depression score on the Center for Epidemiologic Studies Depression Scale (CES-D) was 23 in one study (6) and in another study, 81% of women and 76% of men had a depression score higher than 16 on the CES-D (17). The mean score for depressive symptoms on the Beck Depression Inventory was 29.8 in women and 28.2 in men in another study (24). Mood disorders was not an inclusion criterion in the research study but rather an unselected observation for most studies (20,23,24,28). However, in other studies, participants were only included if there was evidence of psychological distress or criteria for mood or anxiety disorders were met (4,22).

The percentage of participants in individual studies who received a diagnosis of drug and/or alcohol dependence/abuse included (study number in Table 1): 25% (4), 58% lifetime and 9% current (7) 77% (9), 88% (10), 55% (14), 19% and 31% (20), 37% and 10% (22), 20% (23), 31.8%, 53.2%, 18.2%, 36.3%, 33.1% and 18.9% (24), 38.3% (27), 28% (29) and 28% (33). Other studies did not report individual rates but suggested that abused HIV-positive individuals were more likely to have engaged in alcohol or drug abuse and received treatment for substance abuse (2,11-13,15-17,21,26,28,30-32). Drug and/or alcohol dependence/abuse was not an inclusion criteria in the research study but rather an unselected observation for most studies (2,4,7,11-16,20-24,26-33). However, in other studies, participants were only included if there was evidence of drug and/or alcohol dependence/abuse (9,17).

In comparison to the general population (i.e. uninfected and non-abused counterparts), evidence suggests that ongoing risk behaviors and rates of psychopathology are higher in HIV-infected individuals with histories of abuse [7,8,10,31,37,53]. HIV-positive individuals were more likely to report posttraumatic stress, risky health behaviors, substance abuse, chronic stress, and psychiatric history compared with HIV-negative counterparts [53]. In addition, abused individuals reported higher rates of mental illness, compared to non-abused counterparts, suggesting that a history of abuse in childhood increases the likelihood of psychopathology [58]. These findings lend credence to the argument that childhood maltreatment in the context of HIV is worthy of greater attention.

### Intervention studies

The review revealed six intervention studies that have been conducted with this population [8,31-35]. Three of these interventions were carried out in bisexual men and men who have sex with men (MSM) [8,31,35] and three were carried out in mixed samples of males and females [32-34]. A total of 4295 MSM were enrolled into a behavioral intervention trial over 48 months. Behavioral assessments were conducted every 6 months. However, the results revealed that among men reporting a history of CSA, the intervention had no effect in reducing HIV infection rates. Moreover, men reporting a history of CSA were more likely to display depressive symptomatology and use nonprescription drugs [8]. Similarly, 49 gay and bisexual HIV-infected men with histories of CSA were enrolled into an intervention study, consisting of 15 coping group sessions. When compared to an alternative support group intervention and a control condition, the coping group intervention proved to be efficacious in treating HIV-positive adults with histories of CSA. This was attributable to the inclusion of a coping skills training component in the aforementioned treatment condition [31]. Support for the efficacy of the aforementioned coping group intervention was reported in a separate study assessing 28 men and women with HIV and histories of CSA [33]. Similarly findings were reported in another study utilizing the same coping group intervention in 198 HIV-infected men and women with histories of CSA [34]. Reductions in intrusive traumatic stress symptoms were exhibited among participants in the coping group intervention compared to the waitlist condition and in avoidant traumatic stress symptoms compared to the support group condition [34]. Moreover, the efficacy of the aforementioned coping group intervention in reducing sexual transmission risk behavior was assessed [32]. The sexual behavior of 247 HIV-positive men and women with histories of CSA was assessed at baseline, postintervention, and at 4, 8, and 12 month follow-up periods. The frequency of unprotected sexual intercourse for all partners decreased more among participants in the coping group intervention than participants in the support intervention condition [32]. Lastly, a randomized clinical trial comparing the effects of two six-session interventions was carried out in a sample of 137 bisexual men and MSM. Results from both interventions revealed reductions in sexual risk behaviors and number of sexual partners from baseline to posttest, and from 3 to 6 month follow-ups. No significant differences in depression were evident between the two conditions; however, at 6 months the total sample reported a significant decrease in depressive symptoms [35].

### Adherence to antiretroviral medication

In examining mental health outcomes, two articles also reported an association between childhood maltreatment and poor treatment adherence to antiretroviral regimens [25,43]. In one study, a lower percentage of women with a history of physical or sexual abuse reported using Highly Active Antiretroviral Therapies (HAART). Experiencing any physical or sexual abuse increased the likelihood of no HAART use. Women with a history of any physical or sexual abuse were more than 1.5 times more likely to lack HAART, even when clinically eligible [25]. Moreover, the use of HIV medications has been found to be negatively associated with CSA experiences [43].

### High risk behaviors

Many studies found participants commonly reporting engagement in high-risk behaviors such as transactional sex or compulsive sexual behaviors [3,6,39,40,42,53]. Individuals who experienced abuse regularly were more likely to be HIV-positive, exchanged sex for payment, and be a current user of sex-related drugs [40]. It has also been reported that women experiencing CSA were more likely than women without such histories to have used drugs, to have had more than ten sexual partners, to have traded sex for money, drugs, or shelter; and to have been forced to have sex with a person known to be HIV-positive [6]. Moreover, women who had been sexually assaulted were significantly more likely to have shared injection drug equipment [3].

## Discussion

We performed a comprehensive systematic review of the literature to assess mental health outcomes in HIV-positive individuals with histories of childhood maltreatment. To our knowledge, this is the first review of its kind; no published systematic reviews assessing this association have been conducted to date.

The reported mental health outcomes in dually affected individuals (HIV-positive individuals with histories of childhood maltreatment) are in keeping with studies that have investigated these variables separately [11-19,23], supporting at least common outcomes, although assessment of the additive effects of HIV and childhood trauma is difficult in this retrospective review.

The most commonly reported mental illnesses in dually affected individuals included mood, anxiety, and substance abuse disorders. Very few studies examined Axis II disorders. It has been suggested that an HIV diagnosis alone may constitute a significant stressor and thus increase the likelihood of mental illnesses among HIV-positive individuals [59]. Apart from depression or anxiety being a secondary diagnosis to HIV/AIDS, anxiety and depressive symptoms measured over time were also associated with faster progression of the disease after five years. This finding may suggest a reinforcing relationship between HIV and mental illnesses such as depression or anxiety [20]. However, the majority of studies reviewed were cross-sectional in nature, therefore limiting their ability to make causal conclusions around the onset of mental illness in HIV. This highlights the importance of longer term assessment in order to better delineate the nature, severity, and temporal nature of mental health outcomes. Importantly, mental disorders such as depression or anxiety can further impact immune system functioning in HIV and, in turn, influence quality of life and health status [60].

Substance abuse was the most predominant mental health outcome reported in reviewed articles. For the most part, drugs and/or alcohol are used to numb emotional distress and feelings of anger and betrayal resulting from the experience of childhood maltreatment [50]. Not only does substance abuse have direct implications for the progression of the disease in infected individuals [61], it also has direct and indirect implications for the transmission of HIV. Antiretroviral regimens are known to have strong positive effects on quality of life and in improving health status in infected individuals [62]. Few articles have reported an association between childhood maltreatment and poor treatment adherence to antiretroviral regimens or HIV medications [25,43].

Some studies that investigated both early life trauma and adult trauma found an association between childhood trauma and later life trauma [6,28,32,37]. For example, the study by Simoni and Ng found that childhood abuse was correlated with both adult and recent trauma. Moreover, each type of trauma was also correlated with depression scores [49]. Several studies have also found that adult revictimization was very common in survivors of childhood maltreatment [6,32,33,39,50,51]. Further investigation of this relationship and the implications for prevention and intervention is warranted.

HIV-infected men and women may face many current and past negative life events [13] and this may lead to significant adult psychopathology and poor adherence to antiretroviral medications [24,25,63]. In light of this, it is evident that HIV-positive individuals, women in particular, are vulnerable to risk factors associated with abuse, and abuse-related changes in behavioral functioning. These risk factors and behavioral changes may in turn complicate HIV infection.

There are several limitations that warrant mention. First, no search strategy can guarantee the identification of all relevant research, and omission of important studies remains a possibility and may contribute to bias in inferences drawn. Selection or reviewer bias may be a possibility given that studies were not screened or abstracted in duplicate. Second, the heterogeneity across studies presents a problem as it impedes statistical pooling of studies. Third, it is important to note the controversy that abounds in the classification of search terms. There is no standard definition for the experience of abuse as a child. This construct is one that is classified and assessed in a variety of ways. Some studies use broader terms such as childhood adversity or maltreatment, whereas others use more specific terms such as child abuse. For example, in some studies reviewed, childhood maltreatment included diverse types of trauma such as physical abuse and neglect, emotional abuse and neglect, and sexual abuse. Other studies have used more restricted definitions and have only examined CSA or resorted to combining sexual and physical abuse into one category of child abuse. Childhood maltreatment in the present review included emotional, physical, and sexual abuse and neglect. The lack of a standard definition is a further source of bias. Another limitation that warrants mention is that of the design of the review process. The selection criterion for the inclusion of studies in the present review was manuscripts reporting on mental health and childhood maltreatment. However, this should not be mistaken or presented as an argument that childhood maltreatment and impairments in mental health are associated. However, despite these limitations, this review adds substantially to available evidence for both clinical and research decision making.

### Implications for future studies

From the present review, it is clear that very few prospective studies have been executed in this domain [31,44]. The majority of research has been cross-sectional and has included retrospective assessment of childhood maltreatment in HIV-infected individuals. This may be partly due to reasons associated with feasibility and logistics. As cross-sectional study designs preclude follow-up observations and longer term assessment of outcomes, future research should be prospective in nature and should better delineate the nature, severity, and temporal relationship of childhood maltreatment to mental health outcomes and treatment utilization, as well as the mediators and moderators of these outcomes. These studies will allow both clinicians and researchers to better understand the etiology of common mental disorders in HIV-infected samples and reduce bias when making causal inferences. Thus, longitudinal investigation of mental health outcomes in HIV infected individuals with childhood maltreatment will be key to explaining these causal relationships.

## Conclusion

A broad range of adult psychopathology has been reported in studies of HIV-infected individuals with a history of childhood maltreatment. However, a direct causal link cannot be well established. The longer term assessment will better delineate the nature, severity, and temporal relationship of childhood maltreatment to mental health outcomes. There is a need to screen for childhood maltreatment, psychopathology, and associated functioning in HIV-positive individuals and to address these issues in management. Increased focus on the identification and support for children and youth who have experienced childhood maltreatment is necessary. HIV prevention interventions such as education in high-risk behaviors are also a necessity.

## Competing interests

We declare that we have no conflicts of interests.

## Authors’ contributions

GS performed the systematic review and drafted the manuscript. TA, SA, CF-N, JS, and SS participated in its design and coordination and helped to draft the manuscript. All authors read and approved the final manuscript.
